# 9-(Pent-4-en­yl)anthracene

**DOI:** 10.1107/S1600536811030571

**Published:** 2011-08-06

**Authors:** Natarajan Arumugam, Abdulrahman I. Almansour, Usama Karama, Mohd Mustaqim Rosli, Ibrahim Abdul Razak

**Affiliations:** a Department of Chemistry, College of Sciences, King Saud University, PO Box 2455, Riyadh 11451, Saudi Arabia; b X-ray Crystallography Unit, School of Physics, Universiti Sains Malaysia, 11800 USM, Penang, Malaysia

## Abstract

In the title compound, C_19_H_18_, the anthracene system is almost planar, with a maximum deviation of −0.039 (1) Å. The structure is stabilized by C—H⋯π inter­actions. The pentene moiety is not planar and is twisted away from the attached anthracene system with a maximum torsion angle of 91.2 (1)°.

## Related literature

For background to anthracene, see: de Silva *et al.* (1997 ▶); Klarner *et al.* (1998 ▶); Han *et al.* (2009 ▶).
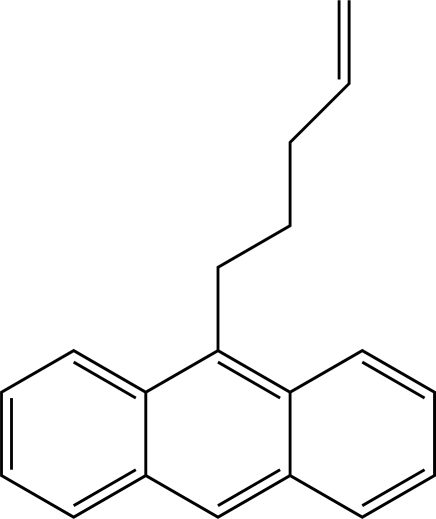


## Experimental

### 

#### Crystal data

C_19_H_18_
*M* *_r_* = 246.33
Monoclinic, 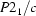

*a* = 11.1555 (2) Å
*b* = 7.2678 (1) Å
*c* = 19.7129 (3) Å
β = 119.096 (1)°
*V* = 1396.55 (4) Å^3^
*Z* = 4
Mo *K*α radiation
μ = 0.07 mm^−1^
*T* = 100 K
0.73 × 0.38 × 0.26 mm

#### Data collection

Bruker SMART APEXII CCD area-detector diffractometer
Absorption correction: multi-scan (*SADABS*; Bruker, 2009 ▶) *T*_min_ = 0.953, *T*_max_ = 0.983
20185 measured reflections
5271 independent reflections
3948 reflections with *I* > 2σ(*I*)
*R*_int_ = 0.028

#### Refinement

*R*[*F* ^2^ > 2σ(*F* ^2^)] = 0.049
*wR*(*F* ^2^) = 0.151
*S* = 1.05
5271 reflections
172 parameters
H-atom parameters constrained
Δρ_max_ = 0.47 e Å^−3^
Δρ_min_ = −0.23 e Å^−3^

### 

Data collection: *APEX2* (Bruker, 2009 ▶); cell refinement: *SAINT* (Bruker, 2009 ▶); data reduction: *SAINT*; program(s) used to solve structure: *SHELXTL* (Sheldrick, 2008 ▶); program(s) used to refine structure: *SHELXTL*; molecular graphics: *SHELXTL*; software used to prepare material for publication: *SHELXTL* and *PLATON* (Spek, 2009 ▶).

## Supplementary Material

Crystal structure: contains datablock(s) I, global. DOI: 10.1107/S1600536811030571/ng5202sup1.cif

Structure factors: contains datablock(s) I. DOI: 10.1107/S1600536811030571/ng5202Isup2.hkl

Supplementary material file. DOI: 10.1107/S1600536811030571/ng5202Isup3.cml

Additional supplementary materials:  crystallographic information; 3D view; checkCIF report

## Figures and Tables

**Table 1:** C—H⋯π interactions (Å, °) *Cg*1 and *Cg*2 are the centroids of the C1–C6 and C1/C6–C8/C13/C14 rings, respectively.

*D*—H⋯*A*	*D*—H	H⋯*A*	*D*⋯*A*	*D*—H⋯*A*
C5—H5*A*⋯*Cg*2^i^	0.95	2.63	3.5729 (9)	175
C7—H7*A*⋯*Cg*1^i^	0.95	2.74	3.6851 (9)	177
C17—H17*A*⋯*Cg*2^ii^	0.99	2.58	3.4643 (9)	149
C18—H18*A*⋯*Cg*1^ii^	0.95	2.90	3.6553 (11)	138

Symmetry codes: (i) 
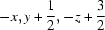
; (ii) 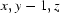.

**Fig. 1.**
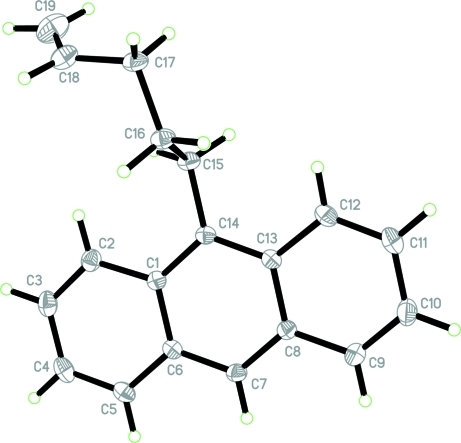
The molecular structure, showing 50% probability displacement ellipsoids. Hydrogen atoms are shown as spheres of arbitrary radius.

## supplementary crystallographic information

### Comment

Anthracene is an attractive material in its photochemical and electrochemical properties as well as used as a potential medium for photoconductive (de Silva *et al.*, 1997) and electroluminescence (Klarner *et al.*, 1998) devices. Furthermore, anthracene derivatives exhibited anticancer activity has also been reported recently (Han *et al.*, 2009). As part of an ongoing study on such compounds, in this paper, we present the crystal structure of the title compound, which was synthesized as an intermediate.

All parameters in (I) within normal ranges. The anthracene is planar with maximum deviation of -0.039 (1)Å from atom C11. In the crystal, C—H···π (Table 1) interactions contribute in stabilizing the crystal structure involving *Cg*1 = C1—C6 and *Cg*2 = C1/C6–8/C13—C14.

### Experimental

A solution of anthrone (1 g, 5.1 mmol) in anhydrous THF (20 ml) was slowly added to pent-4-enylmagnesium bromide (0.47 g, 6.5 mmol). The mixture was stirred for 8 h at room temperature. The reaction mixture was subsequently acidified with 10% HCl, the organic layer was separated, and the aqueous layer was extracted with ether (2 *X* 10 ml). The combined organic layer was washed with water, dried over MgSO_4_ and the solvent was evaporated under reduced pressure and the crude product was added 5 ml benzene, 1.2 g P_2_O_5_ and stirred for 6 h at room temperature. The P_2_O_5_ was filtered off and the benzene was removed under vacuum. The crude product was purified by column chromatography (hexane-dichloromethene 1:1). The product was recrystallized from EtOAc to yield title compound as colourless crystals.

### Refinement

All H-atoms were positioned geometrically and refined using a riding model, with C—H = 0.95 and 0.99 Å, and with *U*_iso_ = 1.2*U*_eq_(C).

### Crystal data

**Table:**

C_19_H_18_	*F*(000) = 528
*M**_r_* = 246.33	*D*_x_ = 1.172 Mg m^−^^3^
Monoclinic, *P*2_1_/*c*	Mo *K*α radiation, λ = 0.71073 Å
Hall symbol: -P 2ybc	Cell parameters from 5794 reflections
*a* = 11.1555 (2) Å	θ = 2.4–33.0°
*b* = 7.2678 (1) Å	µ = 0.07 mm^−^^1^
*c* = 19.7129 (3) Å	*T* = 100 K
β = 119.096 (1)°	Block, colourless
*V* = 1396.55 (4) Å^3^	0.73 × 0.38 × 0.26 mm
*Z* = 4	

### Data collection

**Table:**

Bruker SMART APEXII CCD area-detector diffractometer	5271 independent reflections
Radiation source: fine-focus sealed tube	3948 reflections with *I* > 2σ(*I*)
graphite	*R*_int_ = 0.028
φ and ω scans	θ_max_ = 33.1°, θ_min_ = 2.1°
Absorption correction: multi-scan (*SADABS*; Bruker, 2009)	*h* = −17→16
*T*_min_ = 0.953, *T*_max_ = 0.983	*k* = −9→11
20185 measured reflections	*l* = −30→30

### Refinement

**Table:**

Refinement on *F*^2^	Primary atom site location: structure-invariant direct methods
Least-squares matrix: full	Secondary atom site location: difference Fourier map
*R*[*F*^2^ > 2σ(*F*^2^)] = 0.049	Hydrogen site location: inferred from neighbouring sites
*wR*(*F*^2^) = 0.151	H-atom parameters constrained
*S* = 1.05	*w* = 1/[σ^2^(*F*_o_^2^) + (0.0816*P*)^2^ + 0.1778*P*] where *P* = (*F*_o_^2^ + 2*F*_c_^2^)/3
5271 reflections	(Δ/σ)_max_ = 0.001
172 parameters	Δρ_max_ = 0.47 e Å^−^^3^
0 restraints	Δρ_min_ = −0.23 e Å^−^^3^

### Special details

**Table:**

Experimental. The crystal was placed in the cold stream of an Oxford Cryosystems Cobra open-flow nitrogen cryostat (Cosier & Glazer, 1986) operating at 100.0 (1) K.
Geometry. All e.s.d.'s (except the e.s.d. in the dihedral angle between two l.s. planes) are estimated using the full covariance matrix. The cell e.s.d.'s are taken into account individually in the estimation of e.s.d.'s in distances, angles and torsion angles; correlations between e.s.d.'s in cell parameters are only used when they are defined by crystal symmetry. An approximate (isotropic) treatment of cell e.s.d.'s is used for estimating e.s.d.'s involving l.s. planes.
Refinement. Refinement of *F*^2^ against ALL reflections. The weighted *R*-factor *wR* and goodness of fit *S* are based on *F*^2^, conventional *R*-factors *R* are based on *F*, with *F* set to zero for negative *F*^2^. The threshold expression of *F*^2^ > σ(*F*^2^) is used only for calculating *R*-factors(gt) *etc*. and is not relevant to the choice of reflections for refinement. *R*-factors based on *F*^2^ are statistically about twice as large as those based on *F*, and *R*- factors based on ALL data will be even larger.

### Fractional atomic coordinates and isotropic or equivalent isotropic displacement parameters (Å^2^)

**Table:**

	*x*	*y*	*z*	*U*_iso_*/*U*_eq_	
C1	0.12149 (8)	0.51562 (11)	0.87949 (5)	0.01455 (16)	
C2	0.03754 (9)	0.45215 (12)	0.91135 (5)	0.01904 (17)	
H2A	0.0693	0.3540	0.9478	0.023*	
C3	−0.08723 (9)	0.53006 (14)	0.89033 (5)	0.02260 (19)	
H3A	−0.1405	0.4860	0.9126	0.027*	
C4	−0.13826 (9)	0.67610 (14)	0.83556 (6)	0.02333 (19)	
H4A	−0.2250	0.7294	0.8216	0.028*	
C5	−0.06301 (9)	0.73990 (12)	0.80296 (5)	0.01996 (18)	
H5A	−0.0981	0.8372	0.7662	0.024*	
C6	0.06813 (8)	0.66222 (11)	0.82341 (5)	0.01545 (16)	
C7	0.14534 (8)	0.72875 (11)	0.79022 (5)	0.01635 (16)	
H7A	0.1101	0.8263	0.7536	0.020*	
C8	0.27345 (8)	0.65400 (11)	0.81012 (5)	0.01535 (16)	
C9	0.35206 (9)	0.72202 (13)	0.77582 (5)	0.02136 (18)	
H9A	0.3178	0.8221	0.7404	0.026*	
C10	0.47518 (9)	0.64570 (14)	0.79301 (6)	0.0248 (2)	
H10A	0.5258	0.6919	0.7695	0.030*	
C11	0.52754 (9)	0.49685 (14)	0.84623 (6)	0.02274 (19)	
H11A	0.6129	0.4430	0.8575	0.027*	
C12	0.45707 (8)	0.43008 (12)	0.88137 (5)	0.01865 (17)	
H12A	0.4948	0.3312	0.9172	0.022*	
C13	0.32723 (8)	0.50604 (11)	0.86544 (5)	0.01458 (15)	
C14	0.25201 (8)	0.43960 (11)	0.90096 (5)	0.01431 (15)	
C15	0.30778 (9)	0.28354 (11)	0.95877 (5)	0.01744 (17)	
H15A	0.2711	0.2941	0.9953	0.021*	
H15B	0.4089	0.2943	0.9891	0.021*	
C16	0.27031 (9)	0.09379 (11)	0.91969 (5)	0.01856 (17)	
H16A	0.3180	0.0757	0.8890	0.022*	
H16B	0.1703	0.0887	0.8836	0.022*	
C17	0.31046 (9)	−0.06170 (12)	0.97964 (5)	0.01997 (18)	
H17A	0.3154	−0.1789	0.9555	0.024*	
H17B	0.4028	−0.0361	1.0238	0.024*	
C18	0.21099 (11)	−0.08229 (13)	1.00944 (6)	0.0254 (2)	
H18A	0.1204	−0.1198	0.9734	0.031*	
C19	0.23850 (14)	−0.05277 (16)	1.08162 (7)	0.0363 (3)	
H19A	0.3279	−0.0151	1.1195	0.044*	
H19C	0.1689	−0.0692	1.0957	0.044*	

### Atomic displacement parameters (Å^2^)

**Table:**

	*U*^11^	*U*^22^	*U*^33^	*U*^12^	*U*^13^	*U*^23^
C1	0.0157 (3)	0.0131 (3)	0.0142 (3)	−0.0012 (2)	0.0068 (3)	−0.0018 (3)
C2	0.0204 (4)	0.0205 (4)	0.0175 (4)	−0.0032 (3)	0.0101 (3)	−0.0017 (3)
C3	0.0200 (4)	0.0287 (4)	0.0226 (4)	−0.0048 (3)	0.0131 (3)	−0.0049 (4)
C4	0.0156 (4)	0.0283 (4)	0.0260 (4)	0.0005 (3)	0.0100 (3)	−0.0056 (4)
C5	0.0165 (3)	0.0199 (4)	0.0207 (4)	0.0034 (3)	0.0069 (3)	−0.0005 (3)
C6	0.0146 (3)	0.0147 (3)	0.0157 (3)	0.0005 (3)	0.0063 (3)	−0.0014 (3)
C7	0.0170 (3)	0.0144 (3)	0.0164 (4)	0.0015 (3)	0.0071 (3)	0.0018 (3)
C8	0.0159 (3)	0.0150 (3)	0.0151 (3)	−0.0003 (3)	0.0075 (3)	0.0001 (3)
C9	0.0208 (4)	0.0235 (4)	0.0215 (4)	−0.0012 (3)	0.0116 (3)	0.0027 (3)
C10	0.0209 (4)	0.0321 (5)	0.0253 (4)	−0.0029 (3)	0.0144 (4)	0.0003 (4)
C11	0.0165 (4)	0.0276 (4)	0.0245 (4)	0.0010 (3)	0.0103 (3)	−0.0046 (4)
C12	0.0162 (3)	0.0173 (4)	0.0203 (4)	0.0020 (3)	0.0071 (3)	−0.0017 (3)
C13	0.0141 (3)	0.0132 (3)	0.0152 (3)	0.0003 (2)	0.0061 (3)	−0.0022 (3)
C14	0.0159 (3)	0.0115 (3)	0.0139 (3)	0.0000 (2)	0.0060 (3)	−0.0008 (3)
C15	0.0201 (4)	0.0139 (3)	0.0155 (3)	0.0004 (3)	0.0065 (3)	0.0009 (3)
C16	0.0220 (4)	0.0140 (3)	0.0171 (4)	0.0002 (3)	0.0074 (3)	0.0004 (3)
C17	0.0230 (4)	0.0135 (3)	0.0200 (4)	0.0013 (3)	0.0078 (3)	0.0019 (3)
C18	0.0320 (5)	0.0180 (4)	0.0272 (5)	−0.0009 (3)	0.0151 (4)	0.0021 (3)
C19	0.0531 (7)	0.0295 (5)	0.0336 (6)	0.0003 (5)	0.0267 (5)	0.0015 (5)

### Geometric parameters (Å, °)

**Table:**

C1—C14	1.4159 (11)	C11—C12	1.3650 (13)
C1—C2	1.4340 (11)	C11—H11A	0.9500
C1—C6	1.4393 (11)	C12—C13	1.4349 (11)
C2—C3	1.3670 (12)	C12—H12A	0.9500
C2—H2A	0.9500	C13—C14	1.4139 (11)
C3—C4	1.4208 (14)	C14—C15	1.5108 (11)
C3—H3A	0.9500	C15—C16	1.5352 (11)
C4—C5	1.3628 (13)	C15—H15A	0.9900
C4—H4A	0.9500	C15—H15B	0.9900
C5—C6	1.4311 (11)	C16—C17	1.5361 (12)
C5—H5A	0.9500	C16—H16A	0.9900
C6—C7	1.3971 (12)	C16—H16B	0.9900
C7—C8	1.3950 (11)	C17—C18	1.4941 (14)
C7—H7A	0.9500	C17—H17A	0.9900
C8—C9	1.4309 (12)	C17—H17B	0.9900
C8—C13	1.4385 (11)	C18—C19	1.3193 (15)
C9—C10	1.3625 (13)	C18—H18A	0.9500
C9—H9A	0.9500	C19—H19A	0.9500
C10—C11	1.4199 (14)	C19—H19C	0.9500
C10—H10A	0.9500		
			
C14—C1—C2	122.63 (7)	C11—C12—C13	121.42 (8)
C14—C1—C6	119.97 (7)	C11—C12—H12A	119.3
C2—C1—C6	117.40 (7)	C13—C12—H12A	119.3
C3—C2—C1	121.30 (8)	C14—C13—C12	122.63 (7)
C3—C2—H2A	119.3	C14—C13—C8	120.10 (7)
C1—C2—H2A	119.3	C12—C13—C8	117.26 (7)
C2—C3—C4	120.83 (8)	C13—C14—C1	119.36 (7)
C2—C3—H3A	119.6	C13—C14—C15	120.27 (7)
C4—C3—H3A	119.6	C1—C14—C15	120.32 (7)
C5—C4—C3	120.09 (8)	C14—C15—C16	112.59 (7)
C5—C4—H4A	120.0	C14—C15—H15A	109.1
C3—C4—H4A	120.0	C16—C15—H15A	109.1
C4—C5—C6	120.84 (8)	C14—C15—H15B	109.1
C4—C5—H5A	119.6	C16—C15—H15B	109.1
C6—C5—H5A	119.6	H15A—C15—H15B	107.8
C7—C6—C5	120.67 (8)	C15—C16—C17	111.62 (7)
C7—C6—C1	119.80 (7)	C15—C16—H16A	109.3
C5—C6—C1	119.52 (8)	C17—C16—H16A	109.3
C8—C7—C6	120.93 (7)	C15—C16—H16B	109.3
C8—C7—H7A	119.5	C17—C16—H16B	109.3
C6—C7—H7A	119.5	H16A—C16—H16B	108.0
C7—C8—C9	120.79 (8)	C18—C17—C16	112.44 (7)
C7—C8—C13	119.79 (7)	C18—C17—H17A	109.1
C9—C8—C13	119.41 (7)	C16—C17—H17A	109.1
C10—C9—C8	121.14 (8)	C18—C17—H17B	109.1
C10—C9—H9A	119.4	C16—C17—H17B	109.1
C8—C9—H9A	119.4	H17A—C17—H17B	107.8
C9—C10—C11	119.78 (8)	C19—C18—C17	125.45 (10)
C9—C10—H10A	120.1	C19—C18—H18A	117.3
C11—C10—H10A	120.1	C17—C18—H18A	117.3
C12—C11—C10	120.96 (8)	C18—C19—H19A	120.0
C12—C11—H11A	119.5	C18—C19—H19C	120.0
C10—C11—H11A	119.5	H19A—C19—H19C	120.0
			
C14—C1—C2—C3	179.04 (8)	C11—C12—C13—C14	179.98 (8)
C6—C1—C2—C3	−1.14 (12)	C11—C12—C13—C8	0.79 (12)
C1—C2—C3—C4	0.48 (14)	C7—C8—C13—C14	−1.54 (12)
C2—C3—C4—C5	0.28 (14)	C9—C8—C13—C14	178.80 (8)
C3—C4—C5—C6	−0.31 (14)	C7—C8—C13—C12	177.67 (7)
C4—C5—C6—C7	−179.61 (8)	C9—C8—C13—C12	−1.98 (11)
C4—C5—C6—C1	−0.39 (13)	C12—C13—C14—C1	−176.95 (7)
C14—C1—C6—C7	0.14 (12)	C8—C13—C14—C1	2.22 (12)
C2—C1—C6—C7	−179.68 (7)	C12—C13—C14—C15	0.50 (12)
C14—C1—C6—C5	−179.09 (7)	C8—C13—C14—C15	179.67 (7)
C2—C1—C6—C5	1.09 (12)	C2—C1—C14—C13	178.29 (7)
C5—C6—C7—C8	179.78 (8)	C6—C1—C14—C13	−1.52 (12)
C1—C6—C7—C8	0.56 (12)	C2—C1—C14—C15	0.84 (12)
C6—C7—C8—C9	179.78 (8)	C6—C1—C14—C15	−178.97 (7)
C6—C7—C8—C13	0.13 (12)	C13—C14—C15—C16	−86.19 (9)
C7—C8—C9—C10	−177.80 (8)	C1—C14—C15—C16	91.23 (9)
C13—C8—C9—C10	1.85 (13)	C14—C15—C16—C17	−172.08 (7)
C8—C9—C10—C11	−0.43 (14)	C15—C16—C17—C18	78.26 (9)
C9—C10—C11—C12	−0.83 (15)	C16—C17—C18—C19	−114.87 (11)
C10—C11—C12—C13	0.63 (14)		

### Hydrogen-bond geometry (Å, °)

**Table:**

Cg1 and Cg2 are the centroids of the C1–C6 and C1/C6–C8/C13/C14 rings, respectively.

**Table:**

*D*—H···*A*	*D*—H	H···*A*	*D*···*A*	*D*—H···*A*
C5—H5A···Cg2^i^	0.95	2.63	3.5729 (9)	175
C7—H7A···Cg1^i^	0.95	2.74	3.6851 (9)	177
C17—H17A···Cg2^ii^	0.99	2.58	3.4643 (9)	149
C18—H18A···Cg1^ii^	0.95	2.90	3.6553 (11)	138

Symmetry codes: (i) −*x*, *y*+1/2, −*z*+3/2; (ii) *x*, *y*−1, *z*.
